# Genome-wide characterization of laccase gene family in *Schizophyllum commune* 20R-7-F01, isolated from deep sediment 2 km below the seafloor

**DOI:** 10.3389/fmicb.2022.923451

**Published:** 2022-08-08

**Authors:** Xuan Liu, Muhammad Zain ul Arifeen, Yarong Xue, Changhong Liu

**Affiliations:** State Key Laboratory of Pharmaceutical Biotechnology, Nanjing University, Nanjing, China

**Keywords:** *Schizophyllum commune*, subseafloor, laccase, lignin degradation, fungi, phylogeny

## Abstract

Laccases are ligninolytic enzymes that play a crucial role in various biological processes of filamentous fungi, including fruiting-body formation and lignin degradation. Lignin degradation is a complex process and its degradation in *Schizophyllum commune* is greatly affected by the availability of oxygen. Here, a total of six putative laccase genes (*ScLAC*) were identified from the *S. commune* 20R-7-F01 genome. These genes, which include three typical Cu-oxidase domains, can be classified into three groups based on phylogenetic analysis. *ScLAC* showed distinct intron-exon structures and conserved motifs, suggesting the conservation and diversity of *ScLAC* in gene structures. Additionally, the number and type of cis-acting elements, such as substrate utilization-, stress-, cell division- and transcription activation-related cis-elements, varied between *ScLAC* genes, suggesting that the transcription of laccase genes in *S. commune* 20R-7-F01 could be induced by different substrates, stresses, or other factors. The SNP analysis of resequencing data demonstrated that the *ScLAC* of *S. commune* inhabiting deep subseafloor sediments were significantly different from those of *S. commune* inhabiting terrestrial environments. Similarly, the large variation of conserved motifs number and arrangement of laccase between subseafloor and terrestrial strains indicated that *ScLAC* had a diverse structure. The expression of *ScLAC5* and *ScLAC6* genes was significantly up-regulated in lignin/lignite medium, suggesting that these two laccase genes might be involved in fungal utilization and degradation of lignite and lignin under anaerobic conditions. These findings might help in understanding the function of laccase in white-rot fungi and could provide a scientific basis for further exploring the relationship between the LAC family and anaerobic degradation of lignin by *S. commune*.

## Introduction

Laccase (benzenediol: oxygen oxidoreductase, EC 1.10.3.2) is a metalloprotein belonging to the group of polyphenol oxidases containing copper atoms in the catalytic site and therefore also called blue multicopper oxidases (Baldrian, 2006). Laccase proteins have three conserved domains (Cu-oxidase, Cu-oxidase_2, and Cu-oxidase_3) that are utilized to identify canonical laccases (Bento et al., 2006; Kudanga et al., 2011). Such an arrangement of copper atoms distributed in three domains is present in most of the bacterial and fungal laccases. The catalytic capacity of laccases is actually non-specific but, in most cases, laccases oxidize a range of aromatic compounds, including phenolic moieties typically found in lignin, aromatic amines, benzenothiols, and hydroxyindols as well as non-aromatic compounds, using molecular oxygen as an electron acceptor (Shiba et al., 2000; Claus, 2004; Rodríguez and Toca Herrera, 2006; Chirivì et al., 2012). Laccases have been implicated in a variety of physiological functions in living organisms due to their non-specific catalytic abilities.

Laccase was initially discovered in the Japanese lacquer tree by Yoshida (1883), and since then, it has been found in all domains of life: higher plants, some insects, a few bacteria, and fungi (Solomon et al., 1996; Alexandre and Zhulin, 2000; Claus, 2003). Basic characteristics and functions of laccases are diverse both within and across biological kingdoms. In plants, laccases participate in the radical-based mechanisms of lignin polymer formation (Berthet et al., 2011; Hu et al., 2018), while in fungi, laccases are hypothesized to play a variety of physiological roles, such as stress defense, melanin synthesis (Hua et al., 2018), fruiting-body formation (Lettera et al., 2010; Zhang et al., 2015), and lignin degradation (Singh and Sharma, 2010; Coconi et al., 2018). Lignin degradation is the most important function of fungal laccase. Laccases can directly depolymerize the lignin macromolecule, either alone or in combination with other enzymes. Laccases catalyze the removal of an electron from natural lignin’s phenolic hydroxyl groups, resulting in free phenoxy radicals, and further oxidizes to quinones. Additionally, laccases decarboxylate phenolic and methoxyphenolic acid structures of lignin and cause their demethylation or demethoxylation (Leonowicz et al., 2001). Laccases also are useful biocatalysts for a wide range of biotechnological applications due to their high non-specific oxidation capacity and the use of readily available molecular oxygen as an electron acceptor (Mayer and Staples, 2002). In addition, laccases have important application values in various industrial processes, including textile refining, dye decolorization, bioremediation, lignocellulose delignification, organic synthesis, and food processing (Bilal et al., 2017; Mtibaà et al., 2018; Zhang et al., 2018).

*Schizophyllum commune* belongs to the white rot fungi and is one of the most widely distributed mushrooms on Earth. It is an effective wood-degrading basidiomycete that can produce a large number of hydrolases such as xylanase (Gautam et al., 2018), pectinase (Mehmood et al., 2019), cellulase (Kumar B. et al., 2018), endoglucanase, glycoside hydrolase, and oxidoreductase (Tovar-Herrera et al., 2018). Genome sequencing of a terrestrial strain H4-8 revealed two laccase genes and four genes encoding a distant relative of laccase (Ohm et al., 2010). Our previous study indicated that *S. commune* was the predominant fungal species in deep subseafloor coal-bearing sediments ranging from ∼1.5 to ∼2.0 km below the seafloor (kmbsf), and could grow under both anaerobic and aerobic culture conditions (Liu et al., 2017; Zain Ul Arifeen et al., 2020). Compared with other environmental isolates, strain 20R-7-F01 of *S. commune* isolated from ∼20-million-year-old coal-bearing sediment at 1,966.3 kmbsf has a stronger ability to adapt to *in situ* environmental conditions, including carbon (energy) source, temperature, oxygen, and nitrogen source (Zain Ul Arifeen et al., 2020).

Although laccases were identified and classified in various *S. commune* strains (Kumar et al., 2015, Kumar V. P. et al., 2018; Zhao et al., 2018; Kirtzel et al., 2019), an investigation of the laccase gene family in *S. commune* at the whole-genome level is yet to be conducted. In this study, we identified all possible laccase-coding genes from the *S. commune* reference genome (20R-7-F01). We then analyzed the physical and chemical properties, gene structure, amino acid sequence, systematic evolution, and expression patterns of the gene family in media with or without lignin/lignite. The results could facilitate the understanding of the laccase function in white-rot fungi and provide a scientific basis for further exploring the relationship between the LAC family and the anaerobic degradation of lignin by *S. commune*.

## Materials and methods

### Strains and culture conditions

The fungal strains were isolated from subseafloor sediment, which was collected by drilling vessel at Site C0020 (41°10.5983′N, 142°12.0328′E) in the Pacific Plate off the Shimokita Peninsula, Japan, during the IODP Expedition 337, at a water depth of 1,180 m (Inagaki et al., 2015; Liu et al., 2017). Briefly, the sediment samples were ground into powder in an anaerobic chamber with a flame-sterilized hammer, placed evenly on three petri dishes containing specific media that simulated to the *in situ* environmental conditions, and incubated at 30°C for 7∼14 days (Liu et al., 2017). *S. commune* strains 6R-2-F01, 15R-5-F01, 20R-7-F01, and 24R-3-F01 were obtained from the sediment samples at to 1,496; 1,924; 1,966, and 1,993 mbsf, respectively. Two terrestrial strains CFCC_7252 and CFCC_86625 were purchased from China Forestry Culture Collection Center, which were isolated from Populus wood in Songshan, Beijing and Jurong, Jiangsu of China, respectively. Strain MCCC_3A00233 collected from marine sediment of the Atlantic Ocean was purchased from Third Institute of Oceanography, State Oceanic Administration, People’s Republic of China. and the other five terrestrial strains (225DK, 227DK, MF, Hom2-8, and 207) were obtained from NCBI and JGI database. Details of the habitat and culture conditions of *S. commune* strains have been described previously (Liu et al., 2022). All the fungal strains were maintained on potato dextrose agar (PDA) at 30°C. For DNA and RNA isolations, the fresh mycelia of *S. commune* were inoculated into a 250-ml conical flask containing 150 ml PD (200 g/L potato, 20 g/L glucose) and incubated in a shaking chamber at 30°C, 200 rpm for 7 days.

### Identification of laccase gene family members in *Schizophyllum commune* 20R-7-F01

The *S. commune* 20R-7-F01 genome was assembled using SMRT Analysis and deposited in GenBank under the accession number VCHW00000000. Laccase members contain Cu-oxidase, Cu-oxidase_2, and Cu-oxidase_3 (PF00394, PF07731 and PF07732) domains. The three domains were searched in the *S. commune* 20R-7-F01 genome using HAMMER software (Finn et al., 2011), and protein sequences with three Cu-oxidase domains in the LAC domain were recognized as members of the LAC family. The laccase gene was named using the prefix Sc for *S. commune* followed by the *LAC* gene family abbreviation and numbered sequentially according to their position on unitigs.

### Physical map of *Schizophyllum commune* 20R-7-F01 laccase genes and properties of laccase proteins

Using the *S. commune* 20R-7-F01 genome, the unitig length and the starting position of genes on unitigs were obtained. After statistical analysis, the physical distribution map of their unitigs was visualized using Mapchart 2.32 software (Voorrips, 2002). The theoretical isoelectric point (pI) and molecular weight (MW) of ScLAC proteins were analyzed using the Compute pI/MW tool on the ExPASy server^1^ (Wilkins et al., 1999). Subcellular locations of the ScLAC members were determined using the online software CELLO^2^ (Yu et al., 2006). Signal peptides of each laccase were predicted using SignalP algorithm^3^ (Nielsen et al., 1997). Prediction of transmembrane regions was performed with TMHMM Server^4^ (Krogh et al., 2001). The glycosylation sites of the ScLAC members were predicted by NetNGlyc 1.0^5^ (Gupta and Brunak, 2002).

### Analysis of gene structure and motif composition

The sequence of laccase genes and their coding region were first transformed into FASTA format then matched, and intron/exon structure was determined by comparing the coding sequence of each *ScLAC* gene with its genomic sequence using the Gene Structure Display Server 2.0^6^ (Hu B. et al., 2015). In addition, the upstream regions (1.5 kb) of the *ScLAC* gene sequences were extracted and used for the search of cis-elements using YEASTRACT^7^ (Monteiro et al., 2020). Conserved motifs of laccase proteins were identified statistically using MEME^8^ (Bailey et al., 2009), and the maximum number of motifs to find was set at 10. Visualization of motif compositions was executed with TBtools V1.09 (Chen et al., 2020).

### Sequence alignment and phylogenetic analysis

The identified ScLAC amino acid sequences were aligned separately against each other using ClustalW in MEGA7.0 (Kumar et al., 2016). The conserved regions of ScLAC were used to build the phylogenetic tree. The unrooted phylogenetic tree was created using MEGA7.0 by a neighbor-joining algorithm with bootstrap replication of 100 times. The final phylogenetic tree was visualized and edited in iTOL^9^ (Letunic and Bork, 2016).

### Genome resequencing and variant calling

The genome resequencing and variant detection for *S. commune* strains were carried out according to our previous methods (Liu et al., 2022). Briefly, the genome DNA of *S. commune* strains was extracted and fragmented to generate an approximately 300 bp library insert size and sequenced on an Illumina HiSeq 2500 platform at BGI Genomic (Shenzhen, China). The filtered resequencing reads were mapped to the reference genome of *S. commune* 20R-7-F01 for SNP and variant detection.

### Transcriptome analysis

Total RNA was extracted from mycelia of strain 20R-7-F01 that were cultured in bottles containing lignin and lignite medium, and incubated under anaerobic (i.e., LigWO1-3 and CoalWO1-3) and aerobic (i.e., LigO1-3 and CoalO1-3) condition for seven days (30°C), respectively. Each treatment included three replicates. Lignite was collected from coal mine in Xinjiang. It contains N 1.15%, C 68.7%, H 4.123%, S 1.642%, organic component 98.17%, and inorganic component 1.83%, and vitrinite reflectance was 0.49%. Lignin alkali was purchased from Sigma (CAS# 8068-05-1), which contains 5% moisture. After sampling, all mycelia were immediately frozen in a liquid nitrogen tank and delivered to the Personal Biotechnology Company (Shanghai, China) for mRNA extraction, cDNA library construction, and sequencing. After trimming of low-quality reads (*Q* < 20) and adapter contamination, the clean reads were mapped to the assembled genome of strain 20R-7-F01 using TopHat (Trapnell et al., 2009). Gene prediction was performed using Cufflinks (Roberts et al., 2011). To compare the gene expression level in different libraries, the transcript level of each expressed gene was calculated and normalized to the reads per kilobase of exon model per million mapped reads (RPKM). We used DESeq software for differential analysis of gene expression (Anders and Huber, 2010). Genes with an adjusted *p*-value ≤0.01 and an absolute value of log2 (expression-fold change) ≥1 were deemed to be differentially expressed (Hu L. et al., 2015). The Pheatmap software package in R language was used to perform bidirectional cluster analysis of differential genes and samples. Distances were calculated using the Euclidean method and clustered by complete linkage.

## Results

### Laccase gene family of *Schizophyllum commune* 20R-7-F01

**FIGURE 1:**
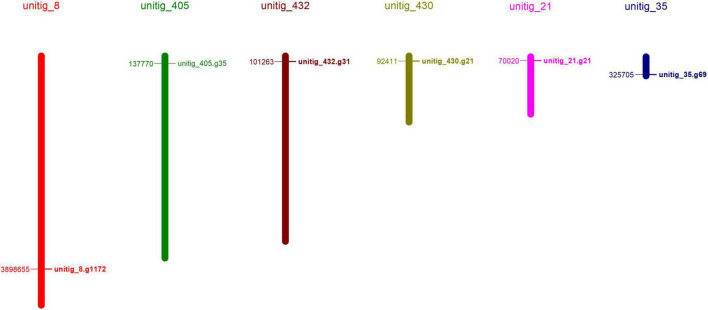
Physical map of the *Schizophyllum commune* 20R-7-F01 laccase genes.

To identify the laccase genes in *S. commune* 20R-7-F01, we searched the genome with HAMMER software for Cu-oxidase, Cu-oxidase_2, and Cu-oxidase_3 domains (PF00394, PF07731, and PF07732). Six putative laccase genes (*ScLAC1* to *ScLAC6*) were identified (Table 1) and mapped to six of the 162 *S. commune* 20R-7-F01 unitigs (Figure 1), indicating that the *ScLAC* gene family did not have the characteristics of tandem replication or clustering.

**TABLE 1:** Basic information of *Schizophyllum commune* laccases.

Gene name	Gene ID	Physical location	AAL (aa)	MW (D)	pI	SL	SP	TR	N-Glyc
ScLAC1	unitig_8.g1172	3898655–3900801	565	62,635.26	4.81	Extracellular	N	N	7
ScLAC2	unitig_405.g35	137770–140761	511	56,942.17	4.62	Cytoplasmic	N	N	4
ScLAC3	unitig_432.g31	101263–107421	1,137	125,249.48	6.55	Nuclear, mitochondrial	N	N	6
ScLAC4	unitig_430.g21	92411–94931	564	61,367.09	5.62	Extracellular	Y	N	5
ScLAC5	unitig_21.g21	70020–72642	651	71,418.18	4.89	Cytoplasmic, extracellular	Y	N	10
ScLAC6	unitig_35.g69	325705–328463	374	41,084.33	6.56	Extracellular	Y	Y	4

### ScLAC proteins

Basic information on all *S. commune* 20R-7-F01 laccases, including gene name, physical location, amino acid length, molecular weight, pI value, subcellular localization, signal peptide and transmembrane topology, were presented in Table 1. The length of laccase proteins ranged from 374 aa (ScLAC6) to 1,137 aa (ScLAC3) residues, and the predicted molecular weights were between 41.08 kDa (ScLAC6) and 125.25 kDa (ScLAC3). The predicted pI-values of the laccase proteins were found to be in the range of 4.62 (ScLAC2) to 6.56 (ScLAC6), indicating that they belonged to acidic proteins. The predicted subcellular locations revealed that the six laccase proteins were located in cytoplasm, nucleus, and mitochondria, and were also found extracellularly. ScLAC1, ScLAC4, and ScLAC6 were predicted to be localized only in the extracellular space, whereas ScLAC3 was located either in the nucleus or mitochondria, ScLAC2 was located in the cytoplasm, and ScLAC5 was located in either the cytoplasm or extracellular. ScLAC4–ScLAC6 were probably signal proteins, while ScLAC1–ScLAC3 may not contain any signal regions. ScLAC6 had transmembrane topology, while the other five laccases did not contain transmembrane domains. Additionally, variable N-glycosylation sites were predicted to be present in all ScLAC proteins (Table 1), indicating that ScLAC family exhibited potential post-translational modifications.

### Gene structure, motif compositions, and phylogeny of *ScLAC*

**FIGURE 2:**
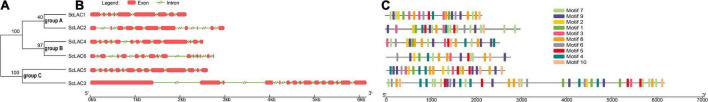
Phylogenetic relationships, gene structure, and motif compositions of the *Schizophyllum commune* 20R-7-F01 laccase gene family. **(A)** A neighbor-joining tree of six ScLAC protein sequences constructed using MEGA v7.0. **(B)** The structure of the six ScLAC genes. Red squares correspond to exons and shrinked green lines indicate introns. **(C)** Schematic motif composition of six ScLAC genes. The colored boxes represent the different motifs, indicated in the top right-hand corner. The scales at the bottom of the image indicate the estimated exon/intron and motif length in kb.

To reveal the structural diversity of *S. commune* 20R-7-F01 laccase genes, we constructed the exon/intron organization and searched for conservative motifs based on the phylogenetic tree of all *S. commune* 20R-7-F01 laccase alignments (Figure 2). Phylogenomic analysis showed that the *S. commune* 20R-7-F01 laccase gene family was clustered into three branches, of which *ScLAC1* and *ScLAC2* were one clade, *ScLAC4* and *ScLAC6* were another clade, and *ScLAC5* and *ScLAC3* were the last clade (Figure 2). In addition, to evaluate the number of laccase genes in the genome of *S. commune* 20R-7-F01, the total number of laccase genes was determined in other Agaricales. The total number of laccase genes varied significantly among species, ranging from 4 in *Hebeloma cylindrosporum* and *Postia placenta* to 55 in *Dendrothele bispora* (Supplementary Figure 1). The amount of laccase in Schizophyllaceae was relatively small compared to other species. In addition, the total number of laccases and protein-coding genes were normalized by genome assembly (in Mb) to avoid potentially misleading comparisons due to differences in genome size and total number of genes among the investigated species. No positive correlation was found between genome size or total number of predicted genes and the number of laccase genes in the corresponding genome (Supplementary Figure 1). For instance, *D. bispora* showed the highest number of laccases (55), but *Moniliophthora perniciosa* had the highest proportion of laccases per total number of genes (0.22%).

The number of introns of *ScLAC* family members varied from 8 to 15. Surprisingly, nearly all of the closest genes on the phylogenetic tree showed remarkably different gene structures. For instance, the introns and exons of *ScLAC5* were most closely arranged, whereas its nearby paralogous gene *ScLAC3* had the longest intron, although their evolutionary relationship reached a 100% bootstrap value. Additionally, *ScLAC6* had the most introns; its coding sequences were divided into 15 parts by introns. In short, *ScLAC3*, *ScLAC5*, and *ScLAC6* were more complicated than the other laccase genes with respect to their structure.

To further reveal the conserved motifs of the ScLAC proteins, we analyzed six ScLAC proteins and identified 10 motifs using the MEME program (Figure 2). As expected, the motif compositions of peer groups had different structures and organizations, which indicated the possibility of functional divergence among those proteins. Although 10 motifs were found in every ScLAC protein, there were some differences in the number of occurrences. For instance, motif-4 was repeated three and six times in ScLAC5 and ScLAC3, respectively. This difference in motif number across ScLAC proteins indicated that different ScLAC proteins may have different functions.

### Cis-regulatory elements predicted in the *ScLAC* promoters

**FIGURE 3:**
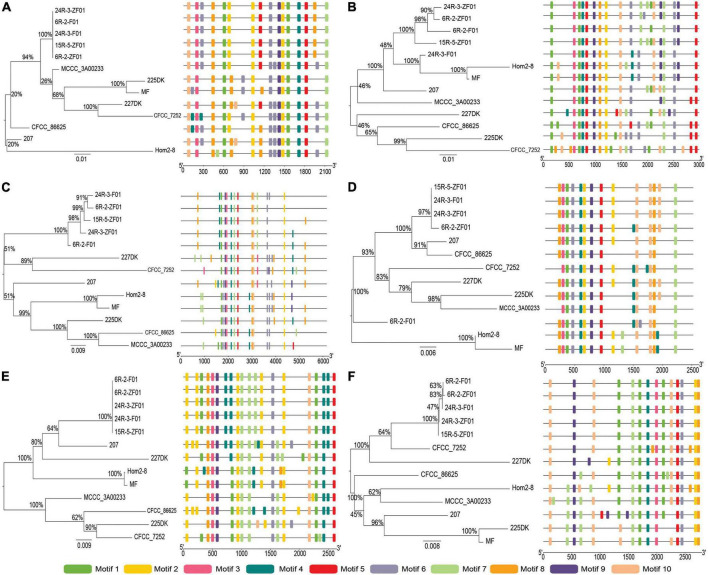
Phylogenetic and motif compositions of the *Schizophyllum commune* population laccase gene family. **(A–F)** Represent neighbor-joining tree of ScLAC1-ScLAC6 protein sequences in *S. commune* population constructed using MEGA v7.0, and schematic motif composition of ScLAC1-ScLAC6 genes in *S. commune* population. The colored boxes represent the different motifs, indicated in the top right-hand corner. The scales at the bottom of the image indicate the motif length in kb.

To obtain further insights into the possible regulatory patterns of ScLAC, we analyzed the cis-acting elements of the 1.5 kb regulatory sequence upstream of the six *ScLAC* gene sequences using the Yeastract database. The promoter regions of *ScLAC1*–*ScLAC6* included various functional cis-acting elements (Figure 3 and Supplementary Table 1) associated with substrate utilization, stress, cell division, and transcription activation. Among them, *ScLAC6* had the most cis-elements, including 16 stress-related, 15 substrate utilization-related, nine cell division-related, and eight amino acid transcription-related cis-elements. In addition, these laccases also contained specific cis-elements; for example, *ScLAC2* contained one specific cis-element, named Nrg2p, which mediated glucose repression and negatively regulated filamentous growth, while *ScLAC3* contained four specific cis-elements, which negatively regulated nitrogen catabolic gene expression and were involved in induction of *CLN3* transcription in response to glucose (Supplementary Figure 2 and Supplementary Table 1). The differences in the number and types of cis-acting elements in *S. commune* 20R-7-F01 suggested that the transcription of laccase genes may be regulated by substrates, stresses, or other factors.

### Laccase differences between subseafloor and terrestrial environments

To understand the evolutionary relationships between subseafloor and terrestrial laccase genes, five subseafloor and eight terrestrial strains were re-sequenced. Phylogenetic analysis based on SNP mutation sites showed that the *ScLAC* of *S. commune* strains inhabiting deep subseafloor sediments differed significantly from that of *S. commune* strains inhabiting terrestrial environments (Figure 3). The average number of SNP mutation sites of the five *ScLAC* in the terrestrial strains was greater than those in subseafloor strains, with the exception of *ScLAC3*, although it contained the most SNP mutation sites (Supplementary Tables 2, 3). In addition, the number and arrangement of conserved motifs in laccases between subseafloor and terrestrial strains also showed various differences, suggesting that *ScLAC* possessed a diverse structure. Among them, the conserved domains of ScLAC2–ScLAC4 were different in both subseafloor and terrestrial strains, indicating that the evolution of these three laccase genes was not only related to habitat but also related to strains (Figure 3).

### Transcriptome analysis of six putative laccase in lignite/lignin degradation

**FIGURE 4:**
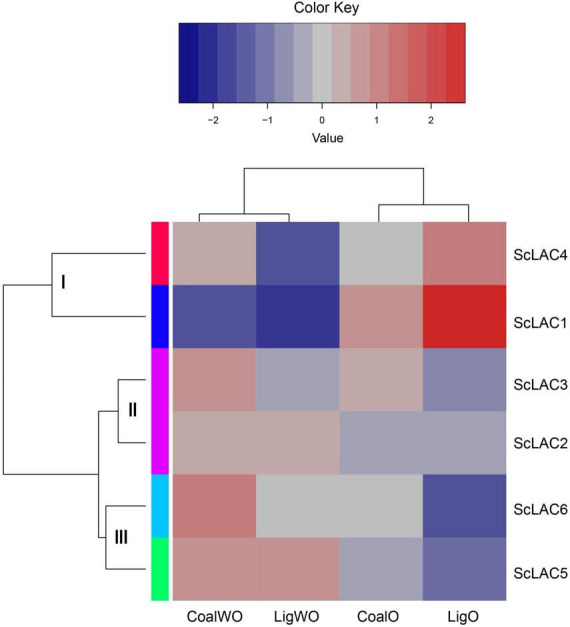
Heatmap of the expression profiles of laccase genes from *Schizophyllum commune* 20R-7-F01 in various carbon sources with or without oxygen. The heatmaps with hierarchical clustering were visualized using the software heatmap2 and the values were log2-transformed with normalization. The blue and red elements indicate low and high relative expression levels, respectively.

RNA-seq analysis of strain 20R-7-F01 cultured in lignin/lignite-containing medium under aerobic and anaerobic conditions at 30°C for 7 days showed that the six laccase genes could be classified into three groups (I, II, and III) (Figure 4). The relative expression levels of *ScLAC1* and *ScLAC4* were lower under anaerobic conditions than under aerobic conditions. In contrast, the laccase genes of group II (*ScLAC2* and *ScLAC3*) and group III (*ScLAC5* and *ScLAC6*) tended to be induced by anaerobic conditions (Figure 4). Additionally, compared with aerobic condition, the expression of *ScLAC5* and *ScLAC6* genes was upregulated by 2.48- and 2.10-fold in the anaerobic conditions (Supplementary Table 4), suggesting that these two laccase genes may be involved in anaerobic utilization and degradation of lignite and lignin by fungi.

## Discussion

Laccases as ligninolytic enzymes play important roles in various biological processes of fungi, including lignin degradation and fruiting-body formation, are typically encoded by gene families (Vasina et al., 2015). Through genome-sequencing analysis, we identified six laccase genes in subseafloor *S. commune* 20R-7-F01, all of which contained three conserved Cu-oxidase domains. However, significant differences were found among these six genes; for instance, very few amino acid sequence similarities were found, and their exon-intron structures were diversified. This suggests that the laccase genes of subseafloor *S. commune* have vast evolutional and functional diversity.

The subcellular localization of proteins is invaluable for understanding their functions and interactions with other proteins (Peng and Gao, 2014). Based on subcellular localization analysis, we found that the ScLAC were located in extracellular, cytoplasmic and nuclear, and mitochondrial. The wide distribution of ScLAC in *S. commune* indicated that these enzymes might have distinct roles in response to various environments (Yang et al., 2021).

The amino acid sequence of fungal laccase generally contains a signal peptide sequence at the N-terminus to guide transmembrane transfer (Jiao et al., 2018). However, some fungal strains have no signal peptide sequence in the laccase gene. For instance, LeLAC3 of *Lentinula edodes* contained a signal peptide sequence in strain D703PP-9, but was absent in strain W1-26 (Sakamoto et al., 2015; Yan et al., 2019). The deficiency of signal peptide sequence was also reported in *Flammulina velutipes* and *Setosphaeria turcica* (Wang et al., 2015; Liu et al., 2019), as well as in plants (Xu et al., 2019). Our study also proved that ScLAC4–ScLAC6 in subseafloor *S. commune* 20R-7-F01 possessed the signal peptide, while ScLAC1–ScLAC3 did not. These data suggest that the laccase genes differ not only between species but also within species.

In fungi, laccase genes differentiate into many paralogous genes and perform various functions throughout the fungal life cycle (Kumar et al., 2003). They are usually clustered in the form of scaffolds; for instance, the 12 laccase genes in *Pleurotus ostreatus* and 13 laccase genes in *L. edodes* were mapped into six and seven scaffolds, respectively (Sakamoto et al., 2015; Jiao et al., 2018). Here, we identified six laccase genes scattered in six unitigs, which were thought to be only specific to *S. commune* 20R-7-F01 genome; similarly, six laccase genes were distributed on five scaffolds of *S. commune* strain H4-8 genome. Therefore, we inferred that this difference might be related to species and strain differences.

Gene structure and protein motif analyses can provide a theoretical basis for the function and classification of laccase family genes (Wang et al., 2019). In general, genes in the same group and subgroup should have a similar conserved domain and motif distribution with closely related members in the phylogenetic tree, revealing the functional similarity between proteins in the same subgroup proteins (Yang et al., 2021). Inconsistent with the results of previous studies, we found that the most closely related members of laccase genes in the phylogenetic tree of *S. commune* had highly diverse motif compositions, and that the conserved motifs of laccases between subseafloor and terrestrial strains were highly diverse. The number and length of introns and exons in *ScLAC* genes were significantly different. In general, groups B and C had more exons and introns than group A (Figure 2). A small number of introns in a gene usually are the result of genetic evolution, which can rapidly regulate genes in response to stress (Stival Sena et al., 2014). Introns are another source of sequence variation (Jacob and Smith, 2017; Naro and Sette, 2017), and intron retention may increase the diversity of proteins and the complexity of genes expression (Zhang et al., 2004).

Cis-elements play significant roles in the regulatory process to respond to multiple abiotic stresses (Feng et al., 2016). The various cis-elements found in the promoter regions of *ScLAC* genes were classified into four major groups: substrate utilization-related, stress-related, cell division-related, and amino acid transcription-related cis-elements. These cis-elements may be recognized by some transcription factors and were thus involved in the regulation and expression of *ScLAC* genes. The presence of multiple cis-elements suggests that *ScLAC* genes could be involved in fungal response to multiple stresses. Laccases are thought to play an important role in fruiting-body formation (Lettera et al., 2010; Zhang et al., 2015) and our recent investigation found that the biosynthesis of amino acids also helps in the formation of fruiting bodies (Zain Ul Arifeen et al., 2021). Thus, the activation of amino acid transcription-related cis-elements in *ScLAC* genes could explain the possible role of *ScLAC* genes in fruiting-body formation.

Lignocellulose degradation by *S. commune* is an important but complex process, which needs to be thoroughly understood. *S. commune* utilizes more than 150 genera of woody plants and can also colonize softwood and grass silage (Ohm et al., 2010). As a model mushroom, *S. commune* H4-8 has complete genome sequence and annotation, and possesses the most extensive polysaccharide decomposition mechanism. The genome of strain H4-8 is rich in the glycoside hydrolase family (hemicellulose and pectin degradation) and polysaccharide lyase family (pectin degradation), which enables it to degrade all plant cell wall components, including lignin (Ohm et al., 2010; Sornlake et al., 2017). Fungi are known to possess a variety of lignin degrading enzymes including lignin peroxidase, manganese peroxidase, dye decolorizing peroxidase, multifunctional peroxidase, and laccase (Floudas et al., 2012). Among these enzymes, laccases are the primary tool lignin degradation in most basidiomycetes (white-rot fungi) and litter-decomposing saprotrophic fungi (Janusz et al., 2020). Laccase catalyzes the one-electron oxidation of substituted phenols, aniline, and aromatic thiols to corresponding free radicals, and reduces molecular oxygen to water (Qi et al., 2015). The broad substrate specificities of laccases, coupled with their use of molecular oxygen as the final electron acceptor rather than the hydrogen peroxide used by ligninolytic peroxidases, makes these enzymes suitable for lignin degradation. However, laccase can only directly degrade phenolic compounds with low-redox-potential, but cannot oxidize the most recalcitrant aromatic hydrocarbons. Nevertheless, some low-molecular-weight compounds produced by fungal degradation of lignin can act as redox mediators to promote the oxidation of refractory substrates (e.g., the non-phenolic lignin moiety) by laccases (Eggert et al., 1996; Camarero et al., 2005).

Basically, laccase use molecular oxygen as the final electron acceptor, and its activity is driven by the concentration of available oxygen (Qi et al., 2015). However, it has been proved that laccase can also oxidize catechol, *o*-aminophenol, *p*-aminophenol, *o*-phenylenediamine, and *p*-phenylenediamine under anaerobic conditions, with activities of 0.978, 0.707, 0.437, 3.603, and 1.039 mg μmol^–1^ min^–1^, respectively (Xie et al., 1999). Shleev et al. (2005) observed direct electron transfer (DET) between the gold electrode and the laccase of *Trametes hirsuta* under anaerobic conditions. Our previous study also proved that laccase may be involved in the anaerobic degradation of phenanthrene by *S. commune* 20R-7-F01 (Zain Ul Arifeen et al., 2022). Based on the transcriptome analysis of *S. commune* 20R-7-F01 during lignin/lignite degradation, we found for the first time that the expression of *ScLAC1* was significantly downregulated under anaerobic conditions, while the expression of *ScLAC5* and *ScLAC6* was significantly up-regulated, compared with that under aerobic conditions (Figure 4 and Supplementary Table 4). These data suggested that *ScLAC5* and *ScLAC6* may play an important role in the utilization of lignite/lignin and other carbon sources by fungi in anaerobic environment. However, the anaerobic catalytic mechanism of laccase and its effect on fungi to obtain nutrition and energy in the anaerobic subseafloor environments need to be further studied.

## Conclusion

A total of six putative laccase genes (*ScLAC*) with three typical Cu-oxidase domains were identified in *S. commune* 20R-7-F01 genome. The physical locations of these genes were mapped in six of 162 unitigs of *S. commune* 20R-7-F01. The theoretical pI of deduced ScLAC proteins widely ranged from 4.62 to 6.56. The MW of the ScLAC proteins ranged from 41.08 to 125.25 kDa and the length varied between 374 and 1,137 amino acids. Based on phylogenetic analysis, the six *ScLAC* genes were classified into three groups with distinct intron-exon structures and conserved motif. All of the ScLAC had cis-elements related to substrate utilization, stress, cell division, and activates transcription of amino acid in the promoter regions, while the number and type of cis-elements had difference between each other. The phylogenetic tree of resequencing data shows that there are many differences in the number and arrangement of conserved motifs between the ScLAC gene of S. commune strains inhabiting deep subseafloor sediments and the ScLAC gene of strains inhabiting terrestrial environments. The expressions of *ScLAC5* and *ScLAC6* genes were significantly upregulation under anaerobic conditions, implying that these two laccase genes might be involved in anaerobic utilization and degradation of lignite and lignin by fungi.

In summary, we identified all possible laccase-coding genes from the *S. commune* reference genome (20R-7-F01) and analyzed the physical and chemical properties, gene structure, amino acid sequence, and systematic evolution, also studied the expression patterns of the gene family under anaerobic and aerobic by growing in lignin/lignite medium. Our data and analysis could facilitate the understanding of the laccase function of white-rot fungi and provide a scientific basis for further exploring the relationship between the *ScLAC* genes family and the anaerobic degradation of lignin by *S. commune*.

## Data availability statement

The datasets presented in this study can be found in online repositories. The names of the repository/repositories and accession number(s) can be found below: https://www.ncbi.nlm.nih.gov/, PRJNA858196; https://www.ncbi.nlm.nih.gov/, PRJNA738972.

## Author contributions

XL performed data analysis and wrote the first draft of the manuscript. MZ helped with data analysis and edited the manuscript. YX cultivated strains of *S. commune* and edited the manuscript. CL conceived the study and edited the manuscript. All authors contributed to the article and approved the submitted version.

## Footnotes

1
http://web.expasy.org/

2
http://cello.life.nctu.edu.tw/

3
https://services.healthtech.dtu.dk/service.php?SignalP-5.0

4
https://services.healthtech.dtu.dk/service.php?TMHMM-2.0

5
https://services.healthtech.dtu.dk/service.php?NetNGlyc-1.0

6
http://gsds.gao-lab.org/index.php

7
http://www.yeastract.com/index.php

8
http://meme-suite.org/tools/meme

9
http://itol.embl.de/

## Supplementary material

Supplementary Figure 1 Comparative analysis of the total number of laccases genes and their distribution across the different subclades in the genome of Agaricales. Percentage of laccases in total number of genes was calculated as following: (total number of laccases/total number of predicted genes in the genome) ×100. Genome size and number of predicted genes were retrieved from NCBI or JGI and refer to the current version of the assembled genome of each species.

Supplementary Figure 2 Venn analysis of predicted cis-elements in the promoter regions of laccase genes from *Schizophyllum commune* 20R-7-F01.
