# Supplementary figures and images for: Genome-wide characterization of laccase gene family in Schizophyllum commune 20R-7-F01, isolated from deep sediment 2 km below the seafloor
Source: Front Microbiol. 2022 Aug 8;13:923451. doi: 10.3389/fmicb.2022.923451 (PMC9393519; doi:10.3389/fmicb.2022.923451)

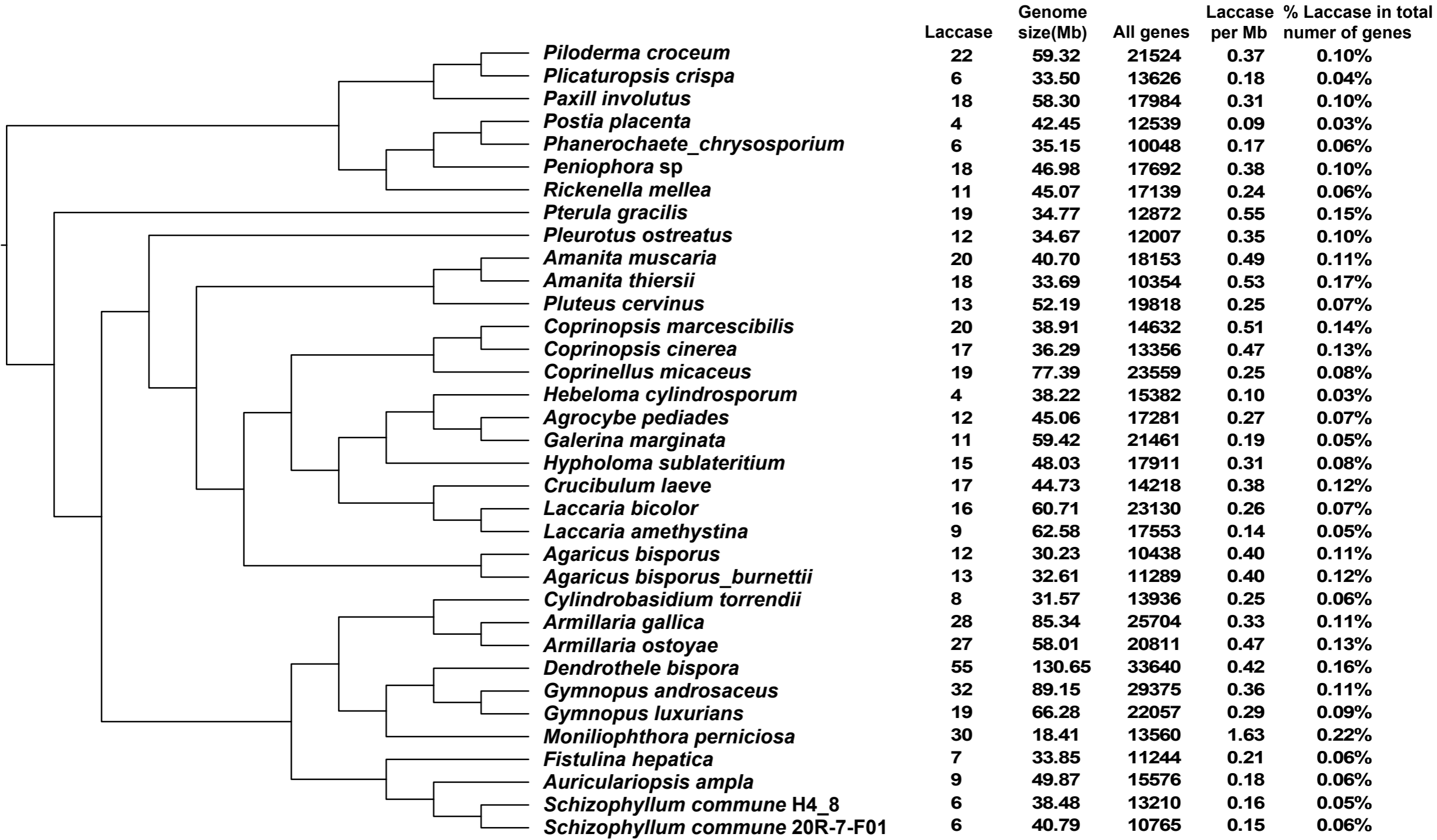

0.08

Supplement: Supplementary Figure 1 — Comparative analysis of the total number of laccases genes and their distribution across the different subclades in the genome of Agaricales. Percentage of laccases in total number of genes was calculated as following: (total number of laccases/total number of predicted genes in the genome) ×100. Genome size and number of predicted genes were retrieved from NCBI or JGI and refer to the current version of the assembled genome of each species. [file Image_1.pdf]

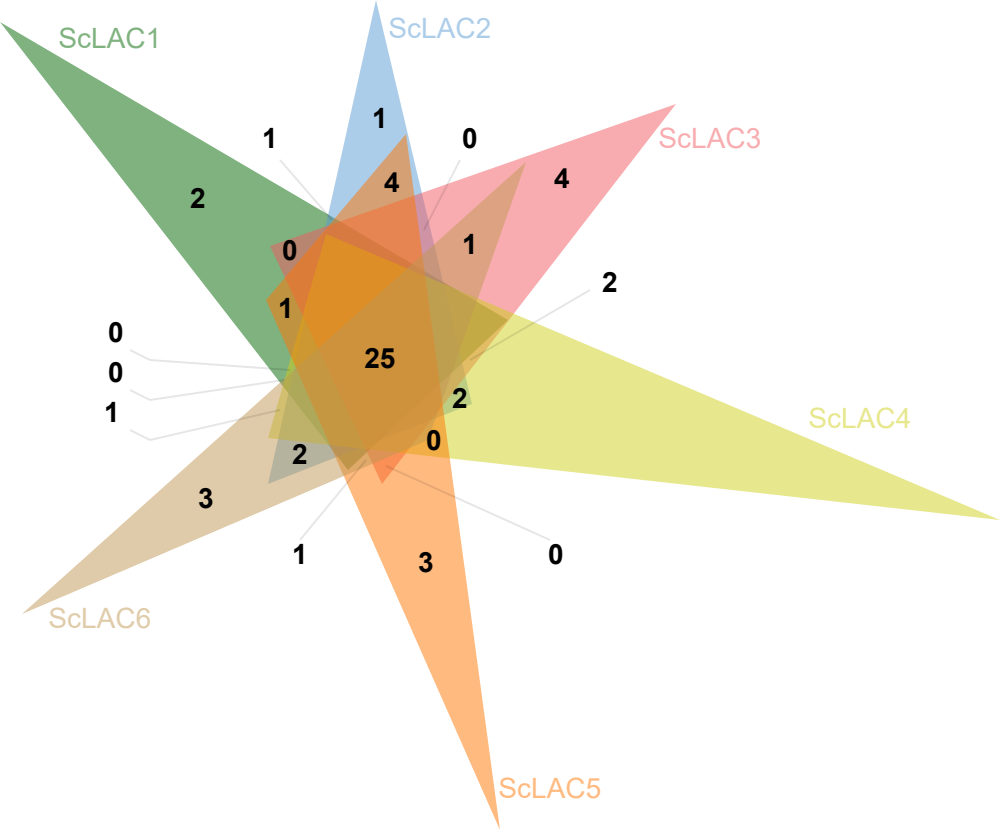

Supplement: Supplementary Figure 2 — Venn analysis of predicted cis-elements in the promoter regions of laccase genes from Schizophyllum commune 20R-7-F01. [file Image_2.pdf]
